# Circulating miRNA-24 and its target YKL-40 as potential biomarkers in patients with coronary heart disease and type 2 diabetes mellitus

**DOI:** 10.18632/oncotarget.18593

**Published:** 2017-06-21

**Authors:** Xin Deng, Yaofang Liu, Mao Luo, Jian Wu, Rongyue Ma, Qin Wan, Jianbo Wu

**Affiliations:** ^1^ Drug Discovery Research Center, Southwest Medical University, Luzhou, Sichuan, China; ^2^ Laboratory for Cardiovascular Pharmacology of Department of Pharmacology, The School of Pharmacy, Southwest Medical University, Luzhou, Sichuan, China; ^3^ Department of Gynaecology and Obstetrics, The Affiliated Hospital of Southwest Medical University, Luzhou, Sichuan, China; ^4^ Medical Research Center, The Affiliated Hospital of Southwest Medical University, Luzhou, Sichuan, China; ^5^ The Lee Woo Sing College, Chinese University of Hong Kong, Sha Tin, Hong Kong; ^6^ Department of Endocrinology, The Affiliated Hospital of Southwest Medical University, Luzhou, Sichuan, China

**Keywords:** biomarker, circulating miR-24, coronary heart disease, YKL-40, type 2 diabetes

## Abstract

Type 2 diabetes mellitus (DM2) is associated with cardiovascular complications and is characterized by high levels of YKL-40, an inflammatory glycoprotein involved in endothelial dysfunction. We investigated the predictive potential of circulating miR-24 in coronary heart diseases (CHD) DM2 patients with CHD, and control subjects. Blood samples were taken from 94 subjects of both genders, and divided over three groups as follows; patients with CHD, patients with DM2 and CHD, and control subjects. Both miR-24 (using real time PCR) and routine parameters were measured. Using bioinformatic analysis and luciferase assays, we found that miR-24 has high complementarity and a high degree of species conservation with respect to the binding sites within the 3′ UTR of the YKL-40 mRNA. The expression levels of circulating miR-24, determined by quantitative real time PCR, were significantly decreased in peripheral blood of DM2-CHD and CHD patients compared with controls. Furthermore, miR-24 strongly associated with DM2-CHD, negatively correlated with YKL-40 in DM2-CHD and DM2 patients after conducting multiple regression analysis. These results provide a novel regulatory mechanism of circulating miR-24 in regulating YKL-40 levels in DM2-CHD, may serve as a biomarker for predicting patients with DM2 and CHD.

## INTRODUCTION

Coronary heart disease (CHD) is a major cause of morbidity and mortality among patients with diabetes mellitus. DM2 is characterized by systemic insulin resistance, which promotes hyperglycemia [1], and it has been proposed that these metabolic abnormalities directly increases the risk of cardiovascular diseases. Endothelial dysfunction plays a key role in determining myocardial infarction in all clinical manifestations of ischemic heart diseases [2, 3]. Previous studies demonstrated that endothelial dysfunction has been linked to DM2 and insulin resistance [4].

YKL-40, also named chitinase 3-like 1 (Chi3l1) [5, 6], produced by a number of different cell types, including: cancer cells, macrophages, neutrophils [7–10], is an inflammatory glycoprotein involved in endothelial dysfunction by regulating chemotaxis, cell attachment and migration, reorganization as a response to endothelial injury [11]. YKL-40 was found to induce coordination of membrane-bound receptor syndecan-1 and integrin αvβ3 and to activate an intracellular signaling cascade, including focal adhesion kinase and MAPK ERK1/2 in endothelial cells [12]. Several studies demonstrated that elevated serum YKL-levels are associated with the presence of coronary artery disease and myocardial infarction [13]. Moreover, elevated serum YKL-40 levels are elevated both in patients with type 1 and type 2 diabetes [14, 15].

Recent studies have shown that miRNA expression profiles can contribute to the development of DM2 and its complications [16]. Circulating miRNAs levels are strongly associated with endothelium activation or damage [17, 18], suggesting that the modification of miRNAs can be developed as novel disease diagnostic or prognostic biomarkers at an early stage in CHD. miR-24 belongs to miR-23~27~24 cluster, and is highly expressed in endothelial cells [19]. During the development of cardiovascular disease, miR-24 plays an important role in regulating endothelial function, such as proliferation, apoptosis, angiopoiesis, inflammation, and differentiation [20]. miR-24 also modulated the TGF-β signaling pathway through targeting gene *FURIN* [21]. However, a relationship between miR-24 and the YKL-40 signaling pathway has not been reported. The current study was carried out to evaluate the change of miR-24 in CHD subjects, and DM2-CHD subjects compared to control ones. In addition, we investigated the correlation between both miR-24 and YKL-40, and the utility of circulating miR-24 as a potential biomarker for predicting DM2-CHD.

## RESULTS

### Clinical data

The study consisted of three subject groups: CHD patients (n=35), DM2 patients with CHD (n=28) and control subjects (n=31). The clinical data of the three subject groups are shown in Supplemental Table 1. In the all subject groups, there was no statistically significant difference in the sex distribution (p>0.05), whereas blood glucose level differed significantly between DM2 patients with CHD compared with CHD patients and controls (p<0.05). There were no significant differences in liver function, including alanine transaminase (ALT), aspartate transaminase (AST) between DM2 patients with CHD compared with CHD patients without chronic complications and controls. Furthermore, Gensini score was significantly higher in DM2 patients with CHD compared to CHD patients and controls (p<0.05). There was also significant difference in Gensini score between CHD patients and those with control subjects (p<0.05). For serum lipids, low-density lipoprotein (LDL), high-density lipoprotein (HDL), total cholesterol (TC), and triglyceride (TG) were no significant difference between DM2 patients with CHD compared with CHD patients and controls. In addition, no significant differences were found in renal function parameters, including serum creatinine (Cr.), blood urea nitrogen (BUN), and uric acid (UA) between DM2 patients with CHD compared with CHD patients and controls.

### Expression levels of circulating miR-24 in DM2 patients with CHD, CHD patients and control subjects

**Figure 1:**
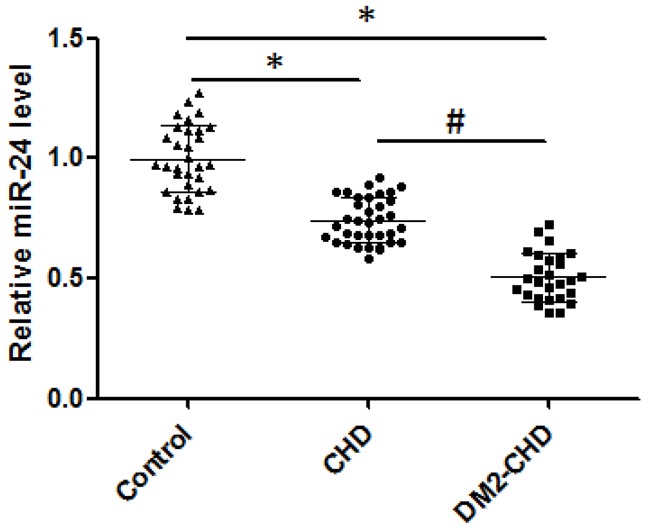
The levels of circulating miR-24 Expression of miR-24 mRNA level was confirmed through qRT-PCR analysis in control, CHD, and DM2-CHD, respectively. The products of qRT-PCR were detected by agarose gel electrophoresis. Total RNA extraction was performed using TRIzol Reagent. The data were normalized to U6 RNA in each sample. The qRT-PCR data were analyzed using the 2 ^−ΔΔCt^ method. *P<0.05, Control *vs* CHD, DM2-CHD; ^#^ *P*<0.05 *vs* CHD group.

The levels of circulating miR-24 were significantly lower in CHD patients than that in control subjects (0.741±0.094 vs. 0.998±0.140; p<0.05). In addition, circulating levels of miR-24 were remarkably decreased in DM2 patients with CHD compared with controls (0.506±0.0101vs. 0.998±0.140; p<0.05) (Figure 1).

### Expression level of circulating YKL-40

**Figure 2:**
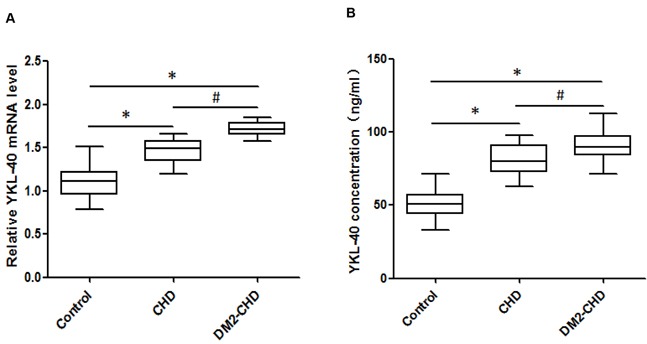
The expression level of circulating YKL-40 **(A)** Expression of YKL-40 mRNA level was confirmed through qRT-PCR analysis in control, CHD, and DM2-CHD, respectively. Total RNA extraction was performed using TRIzol Reagent. The data were normalized to 18S rRNA in each sample. The qRT-PCR data were analyzed using the 2 ^−ΔΔCt^ method. **(B)** YKL-40 levels were determined using ELISA following a BCA assay for PPP (platelet poor plasma) in control, CHD, and DM2-CHD, respectively. *P<0.05, Control *vs* CHD, DM2-CHD; ^#^ *P*<0.05 *vs* CHD group.

YKL-40 mRNA levels were analyzed by qRT-PCR and compared with the level of endogenous genes 18S rRNA. As shown in Figure 2A, the YKL-40 mRNA expression levels were up-regulated and significantly greater in both DM2-CHD and CHD subjects compared with controls, moreover, the levels of YKL-40 mRNA expression were significantly higher in DM2-CHD patients than that in CHD subjects (1.716±0.083 vs. 1.47±0.132; p<0.05). Furthermore, we estimated the total amount of circulating YKL-40 protein antigen by ELISA. As shown in Figure 2B, the average amount of YKL-40 antigen were significantly increased in both DM2-CHD and CHD subjects compared with controls. Higher levels of YKL-40 protein were also found in DM2-CHD patients compared with CHD subjects (91.6±10.0 vs. 80.5±10.6; p<0.05). These results suggested there being increased changes in YKL-40 levels in both DM2-CHD and CHD.

### Prediction analysis

**Figure 3:**
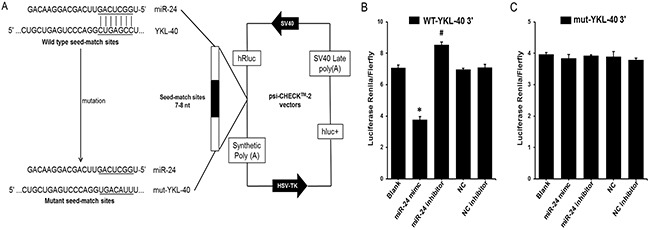
YKL-40 is a direct target of miR-24 **(A)** Schematic representation of the YKL-40 3′ UTR luciferase reporter plasmid. The “seed sequences” and the point mutations in the seed sequences are underlined. **(B)** and **(C)** A miR-24 mimic (50 nmol/L), miR-24 inhibitor (50 nmol/L) or control oligo (50 nmol/L) was co-transfected with the psi-CHECK-2 wild-type or mutated YKL-40 3′ UTR sequence vectors in HEK 293 cells. The relative luciferase activity is reported. All data are presented as the mean ± SEM of triplicate independent experiments.*p < 0.05, miR-24 mimic experimental *vs*. Blank, miR-24 inhibitor experimental, NC and NC inhibitor. ^#^*P* < 0.05, miR-24 inhibitor experimental vs. Blank, miR-24 mimic experimental, NC and NC inhibitor.

Predictions of hsa-miR-24 target genes were performed using a bioinformatics-based approach with Targetscan human software [22], and further confirmed by using the more efficient software miRanda [23], MicroRNA.org [24] and Microcosm [25]. According to the bioinformatics predictions, the software provided information regarding target site accessibility. As shown in Figure 3, there is only a single predicted miR-24 target site in the YKL-40 mRNA 3′ UTR based on good complementarity (ΔG°~ −25.67 kcal/mol), indicating a high degree of site conservation among different mammalian species.

### YKL-40 is a direct target of miR-24

To investigate the predicted interaction of miR-24 with YKL-40 mRNA, the 3′ UTR of human YKL-40 containing the putative miR-24 binding sites was cloned into the psi-CHECK2TM vector downstream of the Renilla luciferase coding sequence and co-transfected with miR-24 mimic, inhibitor or control oligo into HEK 293 cells. An empty vector was used as control (Figure 3A). In the presence of the YKL 3′ UTR, the miR-24 mimic significantly decreased the relative luciferase activity to approximately 50% compared to co-transfection with miR-NC. The relative luciferase activity was significantly increased by the miR-24 inhibitor (Figure 3B). Furthermore, to investigate whether the predicted miR-24 binding sites mediate the effect on YKL-40, miR-24 seed sequences binding to the YKL-40 mRNA 3′ UTR were mutated (Figure 3C). The effects of the miR-24 mimic and inhibitor were abrogated compared to co-transfection of control oligo with vector or empty vector (Figure 3C), suggesting that miR-24 could directly target YKL-40 3′ UTRs.

### Correlation analysis

**Figure 4:**
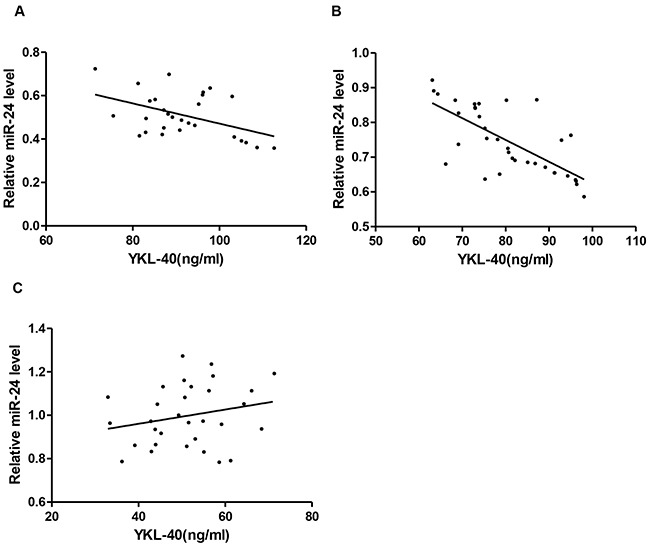
Elevated YKL-40 and reduced miR-24 identified in DM2-CHD, CHD, and control Correlation between circulating miR-24 and plasma YKL-40 in DM2-CHD (**A**), CHD (**B**), and controls (**C**), respectively.

Pearson's coefficient correlation was performed to evaluate the association of circulating miR-24 with YKL-40 level in DM2 patients with CHD, CHD patients and controls, respectively. As shown in Figure 4A-4C, miR-24 was significantly and negatively correlated with YKL-40 in either DM2 patients with CHD (*r* = −0.508, p<0.01) or CHD patients (*r*= −0.703, p<0.001). When these parameters were compared in the control subjects, miR-24 was not significantly correlated with YKL-40 (*r* = 0.227, p>0.05).

### Evaluation of the clinical biomarker

**Figure 5:**
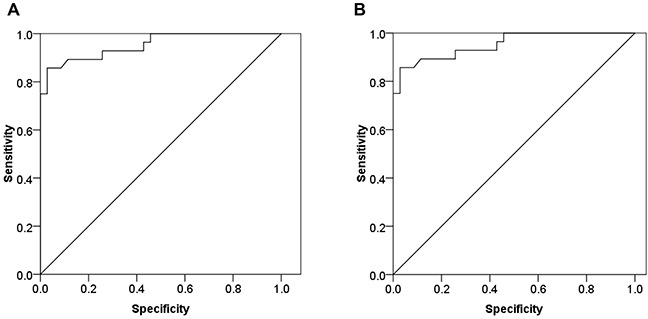
The biomarker potential of circulating miR-24 for DM2-CHD, CHD, and control ROC analysis was used to evaluate the ability of circulating miR-24 to distinguish between different groups. **(A)** MiR-24 distinguished DM2-CHD patients from controls with area under curve (AUC) of 0.975 (Confidence interval [IC]: 0.944~1.0; p<0.001). **(B)** MiR-24 distinguished DM2-CHD patients from CHD patients (AUC: 0.953; CI: 0.899~1.0; p<0.001).

To evaluate if circulating miR-24 could significantly differentiate between DM2 patients with CHD and CHD patients from control subjects. The analysis of ROC curves were constructed and area under the curves (AUCs) were calculated. The ROC curve of miR-24 showed significant ability to discriminate DM2-CHD from CHD patients and control subjects with an AUC of 0.975 (CI: 0.944~1.0; P<0.001), (Figure 5A). MiR-24 also discriminated DM2-CHD patients from CHD with an AUC of 0.953 (CI: 0.899 ~ 1.0; P<0.001) (Figure 5B).

## DISCUSSION

In the current study, we reported a significant reduction in the expression levels of circulating miR-24 in DM2 patients with CHD and CHD patients in comparison to control subjects. These results were further confirmed in multivariate logistic regression analysis which revealed strong association of miR-24 expression with DM2-CHD. We also showed that circulating miR-24 was significantly and negatively correlated with YKL-40 in DM2-CHD, as determined using Pearson's correlation analysis. The findings in our study were that circulating miR-24 discriminated between DM2 patients with CHD, CHD patients, and control subjects, as assessed by ROC analysis.

Circulating YKL-40 levels are elevated both in patients with type 1 and type 2 diabetes, known to be at high risk for the development of cardiovascular diseases and insulin resistance [26]. Several studies demonstrate, that elevated circulating YKL-40 levels are independently associated with the presence and extent of CHD. However, no mechanistic analyses were reported. We now provide that circulating YKL-40 is coordinately regulated by miR-24 in DM2 patients with CHD.

Previous study demonstrated that miRNAs have been reported to regulate vascularity after myocardial infarction [20]. MiRNA profiling has revealed that miR-24 levels were noted to be reduced in DM2 [27]. An increasing number of studies supporting the pivotal role of circulating miRNAs in diabetic patients show that circulating miRNAs may be considered novel biomarkers of cardiovascular diseases [28]. Through bioinformatic analyzing and luciferase assays, we determined miR-24 as directly targeting the 3′UTR of the YKL-40 gene. miR-24 is highly expressed in endothelial cells. We found that miR-24 strongly associated with DM2-CHD, negatively correlated with YKL-40 in DM2-CHD and CHD patients after conducting multiple regression analysis. Our findings lend insight into miR-24-based regulation of YKL-40 and provide a mechanism for the high circulating YKL-40 levels and cardiovascular events observed with DM2.

CHD is responsible for the main proportion of mortality associated with DM2. miR-24 plays an important role in the development of cardiovascular events. While conflicting results have been published regarding the impact of miR-24 on mouse myocardial infarction models [20, 29, 30]. More recently, miR-24 was reported to upregulate vascular endothelial cells von Willebrand factor (VWF) in diabetic patients and correlate with thrombotic event [31]. In line with this study, we identified that decreased miR-24 in plasma is correlated with increased YKL-40 level in DM2 patients with CHD.

Another novel finding, in our results, circulating miR-24 was able to discriminate between DM2 patients with CHD from CHD patients and control subjects with an area under the curve (AUC) of the ROC of 0.975 (CI: 0.944~1.0, P<0.001), suggested that circulating miR-24 exhibited not only a potential prognostic value but also a biomarker for predicting DM2 patients with CHD. We demonstrated that decreased levels of miR-24 is associated with elevated levels of YKL-40 in DM2 patients with CHD. Furthermore, we described a novel regulatory mechanism of miR-24 regulating conserved target YKL-40 expression by directly binding to the YKL-40 mRNA 3′ UTR seed sequence. Our study is to evaluate the possibility of peripheral blood miR-24 as a potential biomarker for DM2 patients with CHD. However, in the present study, the biomarker potential of circulating miR-24 for DM2 patients with CHD was conducted in a relatively small sample size of cases and controls, and thus the utility of miR-24 biomarker will need further evaluation in a large clinical study in future.

## MATERIALS AND METHODS

### Patient selection

All patients provided written informed consent 94 research subjects were Han Chinese individuals who received oral glucose tolerance test (OGTT) and other medical health checkups. Coronary heart disease, in particular acute myocardial infarction (AMI), is the major cause of morbidity and mortality in adults. The patients included in this study sustained an initial CHD event (AMI), other types not included in the group, such as coronary artery bypass graft, percutaneous transluminal angiography or angiographically validated CHD. This study comprised three groups, including 31 control subjects, 35 CHD subjects, and 28 DM-CHD subjects. AMI diagnosis was based on following criteria: (1) chest pain lasting >20 minutes, (2) pathological Q waves or ST-segment elevation/depression on the ECG, and (3) elevation of traditional myocardial markers. All research subjects were received between September 20 and January 20, 2016. Control subjects were not allowed to take aspirin, atorvastatin or nonsteroidal anti-inflammatory drugs for 10 days prior to the investigation. The patient study was conducted in accordance with the Declaration of Helsinki, and the study protocol was approved by the Ethics Committee of Affiliated Hospital of Southwest Medical University. The diagnosis of DM2 was performed according to the World Health Organization criteria, i.e., when fasting, glucose was ≥7 mmol/L (126 mg/dL), the two hour oral glucose tolerance test glucose level was ≥11.1 mmol/L (200 mg/dL) or the subjects had a clinical diagnosis of the disease.

### Bioinformatic analysis

We searched for putative miRNAs that target the 3′ UTR of the YKL-40 mRNA using Targetscan human software32. The results were confirmed using different types of software, including miRanda33, MicroRNA.org.34 and Microcosm35. The evolutionary conservation was studied using Multiple EM for Motif Elicitation (MEME) software (http://meme.nbcr.net/meme/tools/meme).

### Isolation of total RNA and miRNA

Total RNA extraction from blood was performed with TRIzol (Invitrogen, Carlsbad, CA.). Small RNAs, including miRNAs by using the mirVanaTM miRNA Isolation Kit (Ambion, Austin, TX) according to the manufacturer's recommendations. The eluted small RNA was stored at −80°C.

### 3′UTR luciferase reporter gene assay

3′UTR luciferase reporter geneassay was studied as described [32]. A fragment of the YKL-40 mRNA 3′ UTR containing the putative or mutated miR-24 binding site was amplified by RT-PCR from MEG-01 cell total RNA. The products were inserted into the XhoI and NotI restriction sites of thepsi-CHECK2TM vectors (Promega) downstream from the Renilla luciferase coding sequence. The constructs were cotransfected with miR-24 mimic, inhibitor and control oligo into HEK 293 cells using Lipofectamine 2000 (Invitrogen, Grand Island, NY). Cells were harvested after 48 hours of transfection, and firefly luciferase activity was measured using the Dual-Luciferase Reporter Assay System Kit (Promega, E1910) and the luminometer Orion II (Berthold Detection Systems, Germany).

### miR-24 and its target gene YKL-40 quantitative RT-PCR assay

Validation of the expression of the sequence-specific miR-24 was determined using quantitative stem-loop qRT-PCR. Validation of its target gene YKL-40 was performed using qRT-PCR. U6 and 18S rRNA were used as internal controls for normalization. Briefly, small RNAs were transcribed into cDNA using the designed miRNA specific stem loop-RT primers and the NCode™ miRNA First-Strand cDNA Synthesis Kit (Invitrogen). Then, 500 ng of total RNA was reverse transcribed into cDNA using the M-MLV Reverse Transcription Kit (Promega, USA). All qPCRs (20 μl total reaction) were performed with 5 μL of generated cDNA using the SYBR Green Master Mix protocol (TaKaRa Biotechnology Co., Ltd) with the ABI PRISM 7500 system (Applied Biosystems, Forster City, CA, USA). The reactions were incubated in a 96-well plate at 95°C for 10 min, followed by 40 cycles of 95°C for 15 s, 60°C for 30 sec, 72°C for 30 sec. The dissociation curve analysis of the PCR products was determined in the final stage of 55°C to 95°C. All reactions were run in triplicate, and each sample was replicated three times. All fold changes using qRT-PCR were determined using the 2^−ΔΔCT^ mean ± SEM [32, 33]. All primers are listed in Supplementary Table 2.

### Measurement of YKL-40

Blood was collected into citrate anticoagulant, and plasma were prepared by centrifugation. YKL-40 antigen was measured using the human YKL-40 total antigen assay ELISA kit (R&D systems, Minneapolis, MN). The detection limit was 15 μg/L. The intra-assay coefficient of variation (CV) was <5% and the inter-assay CV was <6%. All samples from each patient were analyzed on the same plate to reduce inter-assay CV.

### Statistical analysis

Results were calculated as mean ± standard error of mean (M ± SEM). Pearson χ2 test was used to compare qualitative variables represented as cut off values. Statistical analyses were done using SPSS version 20.0. Multivariate logistic regression analysis was done to determine the variables that independently contributed to the presence of CHD. Discriminating between patients and controls was identified using receiver-operating characteristic (ROC) analysis and area under the curve (AUC) was calculated for circulating miR-24 to assess predictive value. P< 0.05, were considered to be significant.

## SUPPLEMENTARY MATERIALS TABLES


