# Supplementary material for: Circulating miRNA-24 and its target YKL-40 as potential biomarkers in patients with coronary heart disease and type 2 diabetes mellitus
Source: Oncotarget. 2017 Jun 21;8(38):63038–46. doi: 10.18632/oncotarget.18593 (PMC5609901; doi:10.18632/oncotarget.18593)
Supplement: Supplementary file 1 [file oncotarget-08-63038-s001.pdf]

Circulating miRNA-24 and its target YKL-40 as potential biomarkers in patients with coronary heart disease and type 2 diabetes mellitus

SUPPLEMENTARY MATERIALS

Supplementary Table 1: General clinical data

Characteristics	Control	CHD	CHD-DM2
**Number**	31	35	28
**Sex (male/female)**	16(51.6)	18(51.4)	16(57.1)
**Age (years)**	60.3±7.3	61.3±6.6	65.1±7.2
**Hypertension**	15(48.4)	16(45.7)	15(53.6)
**Smoker**	12(38.7)	11(31.4)	9(32.1)
**GLU/(mmol·L^{-1})**	5.11±1.25	4.93±1.12	8.69±1.52[(a,b)]
**ALT/(U·L^{-1})**	20.33±10.21	23.56±9.19	26.55±12.62
**AST/(U·L^{-1})**	21.92±6.98	25.39±7.18	29.14±11.12
**TG(mmol·L^{-1})**	1.15±0.67	1.41±1.02	1.43±1.15
**TC (mmol·L^{-1})**	4.29±0.71	4.28±0.55	4.91±1.06
**LDL-C(mmol·L^{-1})**	1.87±1.38	2.25±0.82	2.11±0.92
**HDL-C(mmol·L^{-1})**	1.05±0.33	1.12±0.29	1.03±0.62
**Cr/(μmol·L^{-1})**	43.30±5.80	41.48±6.81	43.62±6.82
**BUN/(mmol·L^{-1})**	5.16±1.61	5.29±1.22	5.18±1.83
**UA/(μmol·L^{-1})**	346.98±59.81	368.88±62.55	370.91±69.92
**Gensini Score**	0	46.4±9.73[(a)]	64.6±9.64[(a,b)]

Data are presented as number (percentage) for categorical data or mean ± standard error of mean (M ± SEM) for parametrically distributed data. DM2: type 2 diabetes mellitus; CHD: coronary heart disease; GLU: fasting glucose; ALT: alanine transaminase; AST: aspartate transaminase; LDL-C: low-density lipoprotein cholesterol; HDL-C: high-density lipoprotein cholesterol; TC: total cholesterol; TG: triglyceride. Cr: creatinine; BUN: blood urea nitrogen; UA: uric acid. [a]$p<0.05$, [b]$p<0.005$ compared to controls and CHD.

Supplementary Table 2: The sequences of primers used in the study

Stem-loop Q-RT-PCR for miR-24	Stem-loop RT primer of miR-24	5'-CTCAACTGGTGTCGTGGAGTCGGCAATTCA GTTGCCTGCTGA -3'
	Sense primer of miR-24	5'- ACACTCCAGCTGGGTGGCTCAGTTCAGCA GG-3'
	Antisense primer of miR-24	5'- CTCAACTGGTGTCGTGGA -3'
	Sense primer of reference gene U6	5'-CTCGCTTCGGCAGCACA-3'
	Antisense primer of reference gene U6	5'-AACGCTTCACGAATTTGCGT-3'
Real-time PCR for human YKL-40	Sense primer of YKL-40	5'-TGCCCTTGACCGCTCCTCTGTACC-3'
	Antisense primer of YKL-40	5'- GAGCGTCACATCATTCCACTC -3'
	Sense primer of reference gene18S rRNA	5'-CCTGGATACCGCAGCTAGGA-3'
	Antisense primer of reference gene 18S rRNA	5'-GCGGCGCAATACGAATGCCCC-3'
Vector construction	Sense primer of YKL-40 XhoIF	5'-CCGCTCGAGCCCTCTGTTCTGCACACAGCAC GGGGGCCA-3'
	Antisense primer of YKL-40 NotIR	5'-ATAAGAATGCGGCCGCTCACTGTTAAGCTCTT GTACTTTAT-3'
	Sense primer of mut-YKL-40	5'-ATCACCTGCCCTGCTGAGTCCCAGGTGACAT TTCAGTCTCCCTCCCTTGGGGCCTATG -3'
	Antisense primer of mut-YKL-40	5'-CATAGGCCCCAAGGGAGGGAGACTGAAATGTC ACCTGGGACTCAGCAGGGCAGGTGAT-3'
